# The Effect of Low Ambient Relative Humidity on Physical Performance and Perceptual Responses during Load Carriage

**DOI:** 10.3389/fphys.2017.00451

**Published:** 2017-07-06

**Authors:** Igor B. Mekjavic, Ursa Ciuha, Mikael Grönkvist, Ola Eiken

**Affiliations:** ^1^Environmental Physiology Laboratory, Department of Automation, Biocybernetics and Robotics, Jožef Stefan InstituteLjubljana, Slovenia; ^2^Department of Biomedical Physiology and Kinesiology, Simon Fraser UniversityBurnaby, BC, Canada; ^3^Department of Environmental Physiology, School of Health and Technology, Royal Institute of TechnologyStockholm, Sweden

**Keywords:** ambient relative humidity, evaporative cooling, desert conditions

## Abstract

**Introduction:** The study evaluated the effect of low ambient relative humidity on physical performance and perceptual responses during load carriage in a hot environment.

**Methods:** Ten heat-unacclimatized male subjects participated in three 130-min trials, during which they walked on a treadmill, carrying a load of ~35 kg, at a speed of 3.2 km.h^−1^, with an incident wind at the same velocity and ambient temperature at 45°C. Each trial commenced with a 10-min baseline at 20°C and 50% relative humidity (RH), the subjects transferred to a climatic chamber and commenced their simulated hike, comprising two 50-min walks separated by a 20-min rest period. In two, full protective equipment (FP) trials, RH was 10% (partial pressure of water vapor, p_*H*2*O*_ = 7.2 mmHg) in one (FP10), and 20% (p_*H*2*O*_ = 14.4 mmHg; FP20) in the other. In the control trial, subjects were semi-nude (SN) and carried the equipment in their backpacks; RH was 20%. Measurements included oxygen uptake, ventilation, heart rate, rectal and skin temperatures, heat flux, temperature perception, and thermal comfort.

**Results:** In FP20, four subjects terminated the trial prematurely due to signs of heat exhaustion; there were no such signs in FP10 or SN. Upon completion of the trials, pulmonary ventilation, heart rate, and rectal temperature were lower in FP10 (33 ± 5 l/min; 128 ± 21 bpm; 38.2 ± 0.4°C) and SN (34 ± 4 l/min; 113 ± 18 bpm; 38.1 ± 0.4°C than in FP20 (39 ± 8 l/min; 145 ± 12 bpm; 38.6 ± 0.4°C). Evaporation was significantly greater in the SN compared to FP10 and FP20 trials. FP10 was rated thermally more comfortable than FP20.

**Conclusion:** A lower ambient partial pressure of water vapor, reflected in a lower ambient relative humidity, improved cardiorespiratory, thermoregulatory, and perceptual responses during load carriage.

## Introduction

During moderate exercise conducted over a range of ambient temperatures, from 5 to 35°C, Nielsen (1938) demonstrated that the exercise-induced increment in core temperature is not due to the failure of the thermoregulatory system to initiate appropriate heat-loss mechanisms that would prevent the rise in core temperature, but that the increment in core temperature is regulated. He further demonstrated that for a constant intensity of exercise over this range of temperatures, the heat production and total heat loss were constant, but not equal, the difference being reflected in the increase in heat stored in the tissues of the body, resulting in an increase in core temperature, as noted earlier by Dill et al. (1931). The total heat loss was derived from evaporative heat loss, which increased proportionally with the increase in ambient temperature, and convective and radiative heat losses, which decreased in proportion to the increase in ambient temperature. This study demonstrated that the exercise-induced increment in core temperature over a range of ambient temperatures was caused by the external work intensity. The relative humidity in these experiments was not regulated, and ranged between 35 and 55%.

A higher ambient relative humidity in these experiments would have resulted in a lower partial pressure of water vapor gradient between the skin and ambient air, and thus at some critical gradient a reduced capacity to evaporate sweat (Berglund and Gonzalez, 1977). This critical partial pressure of water vapor gradient delineates the ambient conditions in which the body can compensate for the increase in heat storage, by losing heat through the heat loss pathways of conduction, convection, radiation and evaporation (compensable ambient conditions), from those ambient conditions in which it cannot (uncompensable ambient conditions). The resultant greater difference between the heat produced by exercise and that lost to the ambient would have mediated a greater increase in core temperature, resulting in a reduced exercise capacity. The reduction in exercise capacity with increasing relative humidity was nicely demonstrated by Maughan et al. (2012) for high exercise intensities, and by Moyen et al. (2014a) for low exercise intensities. Maughan et al. (2012) reported that elevated ambient humidity caused a significant reduction in time to exhaustion of subjects exercising on a cycle ergometer at an ambient temperature of 30°C with relative humidity ranging from 24 to 80%. Moyen et al. (2014a) concluded that at lower exercise intensities conducted at ambient temperatures of 35°C, evaporative heat loss decreased at an ambient relative humidity of 55%, and that non-evaporative and respiratory heat loss had a negligible effect on heat balance. In both studies, higher ambient relative humidity caused a greater increase in core temperature.

For the low exercise intensities used in their study, Moyen et al. (2014a) proposed the existence of a threshold ambient relative humidity somewhere in the range of 55 to 70% at an ambient temperature of 35°C, above which thermal strain (defined in the Glossary of terms for thermal physiology as any deviation of body temperature induced by sustained thermal stress that cannot be fully compensated by temperature regulation; Simon, 1987) is to be anticipated. This was later confirmed in female subjects (Moyen et al., 2014b). The results of Moyen et al. on exercising male (Moyen et al., 2014a) and female (Moyen et al., 2014b) subjects confirmed the earlier findings in resting human subjects of Bebner et al. (1958), who concluded that below a critical ambient humidity, when the skin is not completely wet, the rate of evaporative heat loss depends predominantly on the rate of sweating resulting from the metabolic rate and ambient heat load. Bebner et al. (1958) determined this critical relative humidity to be 60% at 40°C (p_H2O_ = 33.2 mmHg), and 83% at 36°C (p_H2O_ = 37.0 mmHg). In both of these steady state ambient temperature conditions, sweat rate did not vary to any significant degree below these critical humidities. It was independent of the ambient temperature, and evaporative heat loss was dependent on the rate sweat was secreted on the skin surface. In contrast, above these critical ambient relative humidities, sweat rate increased substantially with increasing relative humidity. Thus, once skin is completely wetted, the evaporation of sweat from the skin surface is more a function of the ambient humidity than the actual rate of sweating. As a consequence, a lower ambient partial pressure of water vapor, reflected in a lower ambient relative humidity, should provide improvement in performance. The test of this hypothesis was the aim of the present study, in which we compared the thermoregulatory responses and performance of subjects carrying an identical load on a treadmill at an ambient temperature of 45°C, between conditions where the ambient relative humidity was maintained at either 10% (P_H2O_ = 7.2 mmHg) or 20% (P_H2O_ = 14.4 mmHg). We reasoned that the identical external work rate in the two conditions would result in the same heat production. Any differences in body heat storage, either due to differences in sweating rate and/or evaporation would be reflected in the core temperature and subjects' responses during the conduct of the task. In addition to these two trials during which subjects wore the same protective clothing, a control trial was also completed at 45°C and 20% relative humidity, in which the subjects carried the same load, but were semi-naked. Comparison of the results of this control trial with those of the trial in which subjects wore protective clothing (20%RH at 45°C) would reveal the benefit, if any, of the clothing layer.

## Methods

Ten healthy heat-unacclimatized male volunteers participated in the study (Table 1). They were all physically active and participated in at least one physical activity several times per week. The study was performed in a climatic chamber (IZR d.d., Skofja Loka, Slovenia) and the protocol of the study approved by the National Committee for Medical Ethics at the Ministry of Health (Republic of Slovenia). Subjects were informed about the experimental protocol before giving their consent to participate in the study, and were instructed that they could terminate any given trial at any time, and were also free to withdraw from the study at any time.

**Table 1:** Subjects' physical characteristics.

**Subject**	**Age**	**Weight**	**Height**
S1	24	91.4	176
S2	26	76.3	169
S3	22	68.1	172
S4	27	68.9	196
S5	28	71.6	179
S6	28	77.1	170
S7	22	76.3	172
S8	30	88.7	190
S9	25	67.4	177
S10	22	73.3	177
Average	25	75.9	178
*SD*	3	8.3	9

### Experimental protocol

Subjects were requested not to eat or drink coffee 3 h prior to a trial, and not to participate in any heavy exercise during the entire study. Before commencing the trial, subjects were encouraged to drink water, but their hydration level was not measured prior to testing. For each subject, all trials were carried out at the same time of day, with at least 3 days of rest between trials, to avoid effects of fatigue and circadian influences on the measured responses. The trials were randomized.

Once instrumented with sensors and fully equipped, the subject rested outside the chamber for 10 min [ambient temperature (Ta) = 25°C, ambient relative humidity (RH) = 40%], and baseline measurements were obtained. Thereafter, the subject entered the climatic chamber, where ambient conditions were maintained at: Ta = 45°C, RH = 10, or 20%, wind speed and treadmill speed = 3.2 km/h. In each trial, the subject completed two 50-min walk cycles, separated by a 20-min period of rest, during which the treadmill and fans were switched off, and the subject was placed on a chair that also provided support for the backpack.

During the 20-min rest phase, the subject drank 0.5 l of water, previously conditioned in the chamber, such that the temperature of the water was the same as the temperature of the air in the climatic chamber (45°C). The total amount of sweat produced during each trial was calculated from the difference in nude body weight before and after the trial, corrected for water intake. The subjects' weight was also recorded at the beginning and the end of the rest period. Sweat absorbed by the clothing was calculated from the difference in the weight of each clothing item before and after the trial. The trial continued until rectal temperature (Tre) reached 39.0°C, or until the subject attained his individual predicted maximum heart rate (HR_max_ = 220–age), and/or when subjective tolerance limits were noted (exhaustion, dizziness, nausea, or other subjective complaints).

### Clothing and load carriage during the trials

In both full protective (FP) equipment trials [FP in 10% RH (FP10); FP in 20% RH (FP20)] subjects wore: underpants, t-shirt, combat shirt, combat jacket, combat trousers, body armor, combat vest, backpack, helmet, gloves, socks, boots, and carried a dummy rifle. Using a sweating thermal manikin (Jozef Stefan Institute, Ljubljana, Slovenia) the thermal resistance of the clothing ensemble was determined to be 0.56 K.m^2^.W^−1^ (3.62 CLO). The evaporative rate was 1.69 g.min^−1^.m^−2^ at 10% ambient relative humidity and 1.32 g.min^−1^.m^−2^ at 20% ambient relative humidity. As a Control trial, a semi-nude condition (SN) was also performed at 45°C and 20% RH with subjects carrying all the equipment in the backpack, except the boots, shorts, and dummy rifle. During the rest periods, subjects sat on a chair, with the weight of the backpack supported. The total weight of the clothing and equipment were similar for all subjects (~35 kg); any slight differences in the weights were due to different shoe and clothing sizes.

### Equipment

Each subject walked on a treadmill (Woodway Model PPS Med treadmill, Woodway GmbH, Weil am Rein, Germany) with a walking area of 56 × 173 cm. To simulate a walk-dependant headwind, two fans with wing diameters of 85 cm (Biomed d.o.o., Ljubljana, Slovenia) were positioned at a distance of about 80 cm in front of the treadmill. A constant laminar flow of air from the fans to the walking surface of the treadmill to a height of ~200 cm above the treadmill in a straight head-ward direction was achieved by placing a frame containing 36 pieces of PVC tubing, each with an inner diameter of 15 cm, and length of 50 cm, in front of the fans. Wind velocity at each tube was then measured with an anemometer. The tubes were arranged to completely cover the area of the two fans, and to direct the air flow from the fans toward the treadmill. Skin temperature (Tsk) and heat flux (Q) were measured at minute intervals, at 12 different sites of the body with combined Tsk/Q sensors, and the data were sampled and stored with a data logger Almemo Model 5990-2 data-acquisition system (Ahlborn GmbH, Holzkirchen, Germany). Sensors were positioned on the foot, calf, front thigh, back thigh, abdomen, chest, lower back, upper back, upper arm, forearm, palm, and forehead, on the right side of the body. The assessment of Tsk and Q enabled the calculation of unweighted mean Tsk and Q for the overall body. Core Tre was assessed with a rectal thermistor (MSR Electronics GmbH, Henggart, Switzerland), inserted 12 cm beyond the anal sphincter. An Almemo 2590-9 weather station (Ahlborn, Holzkirchen, Germany) provided information about Ta and RH in the chamber during the trials. Subjects' weight and the weight of the heavier (>5 kg) clothing items, such as body armor and backpack, were measured with a model TPT 5N Libela Elsi (Celje, Slovenia) weight scale, with range and resolution of 300 ± 0.25 kg. The rest of the clothing items, weighing <5 kg, were weighed on a model UWE HGM-4000, Universal Weight Enterprises (Hsin Tien City, Taiwan) weight scale, with range and resolution of 4,000 ± 0.2 g. Oxygen uptake (VO_2_; ml/min), and expired minute ventilation (V_E_; ml/min) were monitored with a COSMED K4b2 system (COSMED Srl, Pavona di Albano, Rome, Italy), with measurements obtained during the 10-min rest period outside the climatic chamber, and during the first 5 and the last 5 min of each walking phase. The last minute of each recording was averaged and used for analysis.

During trials, subjects evaluated their perception of thermal comfort (TC), wear comfort (WC), thermal sensation (TS), and perceived exertion (RPE). They provided ratings of TC and WC from a seven-point scale, with the assigned descriptors being: 0–0.5 = comfortable; 1–1.5 = slightly uncomfortable; 2–2.5 = uncomfortable; and 3 = very uncomfortable. Similarly, they provided ratings of TS on a seven-point scale, with the following descriptors: 3 = hot; 2 = moderately hot; 1 = warm; 0 = neutral; −1 = cool; −2 = moderately cold; and −3 = cold. RPE was rated using the 15-point Borg scale (ratings ranging from 6 to 20) for the whole body: 6–7 = very very light; 8–9 = very light; 10–11 = fairy light; 12–13 = somewhat hard; 14–15 = hard; 16–17 = very hard; and 18–19 = very very hard (Borg, 1970). The first ratings were provided outside the chamber during the baseline measurements. Subjects were asked to provide the following ratings after 15-min of walking, after 30-min and again after 45-min of walking. They also provided ratings at the beginning of the rest period, and a few minutes before finishing the rest period.

### Statistical analysis

Statistical significance of differences for temperature measurements (Tre, Tsk, and Q), HR, metabolic variables (VO_2_, V_*E*_), and ambient conditions over time and between experimental trials were calculated with two-way repeated measures ANOVA (experimental trial × time). If a significant *F*-value was found (*p* < 0.05), critical differences were analyzed by Tukey's procedure to locate the significant mean differences. Differences in sweat production and evaporation and the final end-point between experimental trials were compared using paired-samples *t*-test. For the subjectively rated variables, differences were evaluated using the non-parametric Wilcoxon matched-pairs test, with *p* < 0.05 regarded as statistically significant. Measurements, recorded at minute intervals, are presented as means ± *SD*, unless otherwise stated. Subjective ratings are presented as medians (ranges). Statistical analysis was performed using SPSS (version 20, Chicago, IL, USA).

Since all subjects, who terminated a trial prematurely in the FP20 trial, were heat exhausted, evident also in the obtained measurements, it was assumed that they provided a maximum effort. Therefore, for each subject, the termination time point in the experimental trial of shortest duration (invariably the FP20 trial) determined the final time point for comparison between experimental trials. Values obtained at the final time point in the shortest trial, henceforth termed “final time point,” were compared with corresponding time-point values obtained in the other trials, henceforth termed “final values.”

## Results

There were no statistically significant differences between baseline values of the measured thermoregulatory responses (Tre, Tsk, Q), cardiorespiratory responses (HR, VO_2_, V_E_), and subjective ratings (TC, WC, TS) between FP10 and FP20. Comparing the FP conditions (FP10 and FP20) to the SN resulted in similar measured Tre and HR, but lower Tsk and higher Q in the latter during baseline measurements.

Four subjects were not able to complete the FP20 trial due to headache, nausea, dizziness, and paraesthesia in the arms. Three of these subjects managed to complete the first walk and rest, but withdrew during the second walk, while one of the subjects did not manage to complete the rest period. In the FP10, only one subject was unable to complete the trial due to shoulder pain, whereas all subjects completed the SN trial. Since only six subjects were able to complete the FP20 condition, this was taken into account when analyzing and interpreting the data (details explained in Statistical Analysis Section).

### Ambient temperature (Ta, °C) and relative humidity (RH, %)

Ambient temperature did not differ between experimental conditions with 45.2 ± 0.6°C in FP10, 45.3 ± 0.4°C in FP20, and 45.5 ± 0.3°C in SN. As part of the study design comparing different ambient RH, the average ambient RH was 11.1 ± 0.9% in FP10, 22.5 ± 0.7% in FP20, and 22.5 ± 0.2% in SN.

### Rectal temperature (Tre, °C)

**Figure 1:**
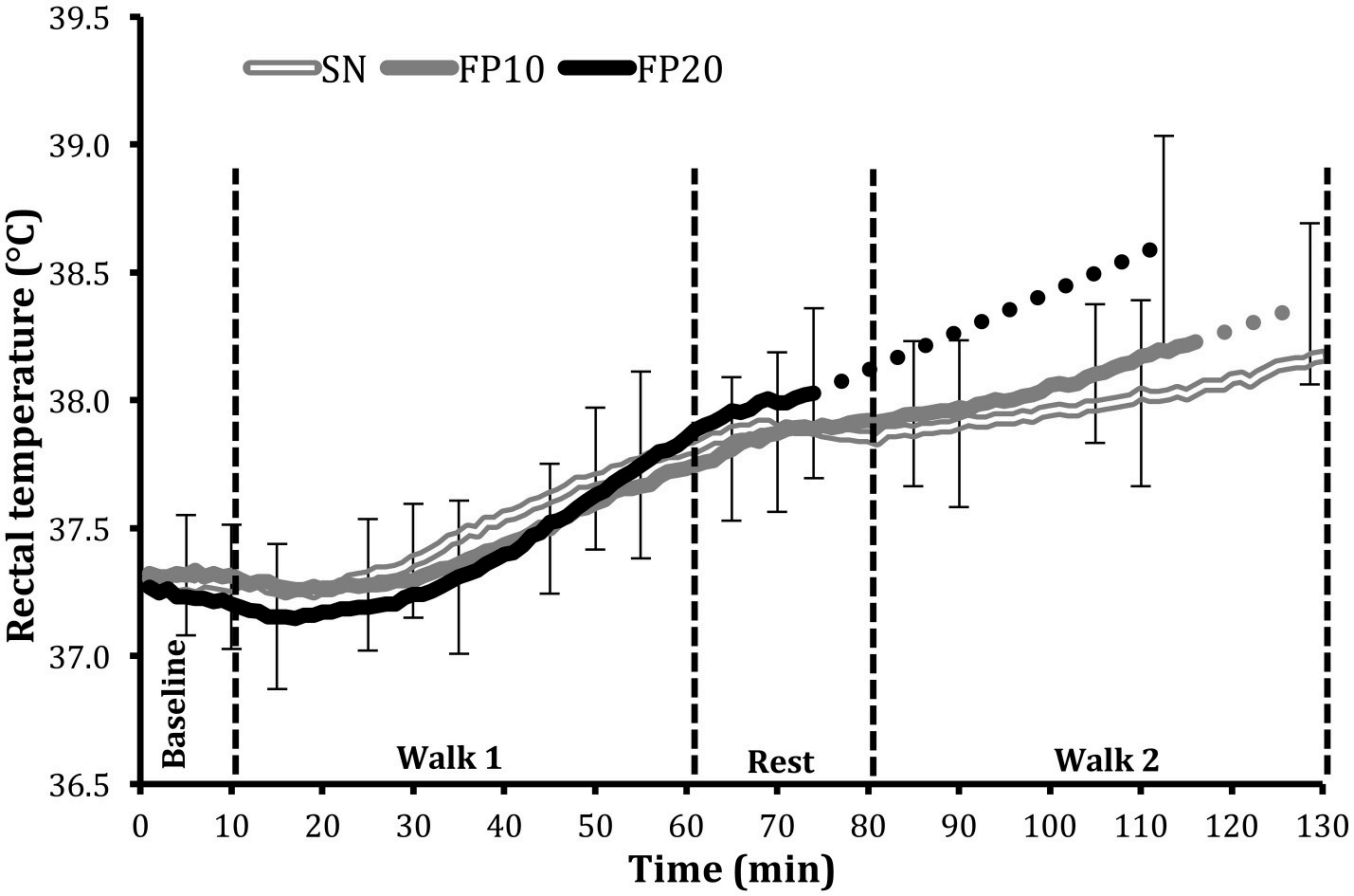
Mean core temperature (°C) ± *SD* during the full protective equipment trials (FP), performed at 10% (FP10), and 20% (FP20) ambient relative humidity, and during semi-nude condition (SN). For the two conditions where subjects terminated the trial prematurely (FP10 and FP20), the last data point for these subjects was averaged with the final data point, obtained from the subjects completing the trial. The solid line presents all 10 subjects' data whereas the dotted lines denote interpolations of the remaining portions of the responses up to the final data point.

Comparison of the results of the first 65 min of the trials, including all subjects' data before early terminations (Figure 1), indicated no difference in Tre between experimental trials with mean trial values of 37.5 ± 0.3°C in FP10, 37.5 ± 0.3°C in FP20, and 37.6 ± 0.2°C in SN (*p* = 0.63). The final time point for all subjects (see also Section Statistical analysis), however, indicated higher Tre in FP20 (38.6 ± 0.4°C) than in FP10 (38.2 ± 0.4°C; *p* < 0.001) and SN (38.1 ± 0.4°C; *p* < 0.001).

### Heart rate (HR, min^−1^)

**Figure 2:**
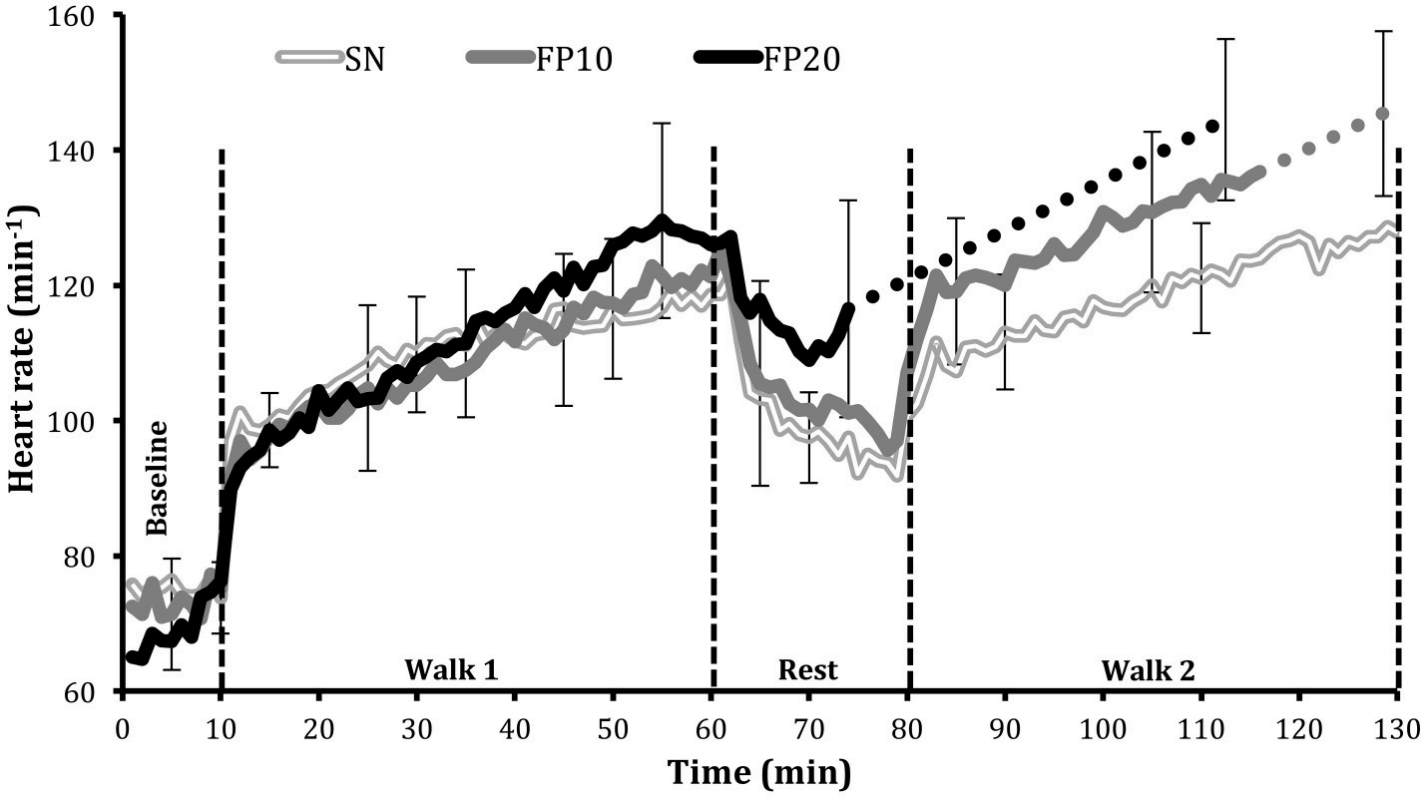
Mean heart rate (bpm) ± *SD* during the full protective equipment trials (FP), performed at 10% (FP10), and 20% (FP20) ambient relative humidity, and during semi-nude condition (SN). For the two conditions where subjects terminated the trial prematurely (FP10 and FP20), the last data point for these subjects was averaged with the final data point, obtained from the subjects completing the trial. The solid line presents all 10 subjects' data whereas the dotted lines denote interpolations of the remaining portions of the responses up to the final data point.

During the first walk and rest (Figure 2), HR did not differ between FP10 (109 ± 11 bpm), FP20 (113 ± 10 bpm), and SN (109 ± 7 bpm; *p* = 0.19). At the final time point, however, there were significant differences between all three experimental trials (FP10 vs. FP20: *p* < 0.05; FP10 vs. SN: *p* < 0.01; FP20 vs. SN: *p* < 0.001), with the lowest HR measured in SN (113 ± 18 bpm), followed by FP10 (128 ± 21 bpm), and finally FP20 (145 ± 12 bpm).

### Skin temperature (Tsk, °C)

**Figure 3:**
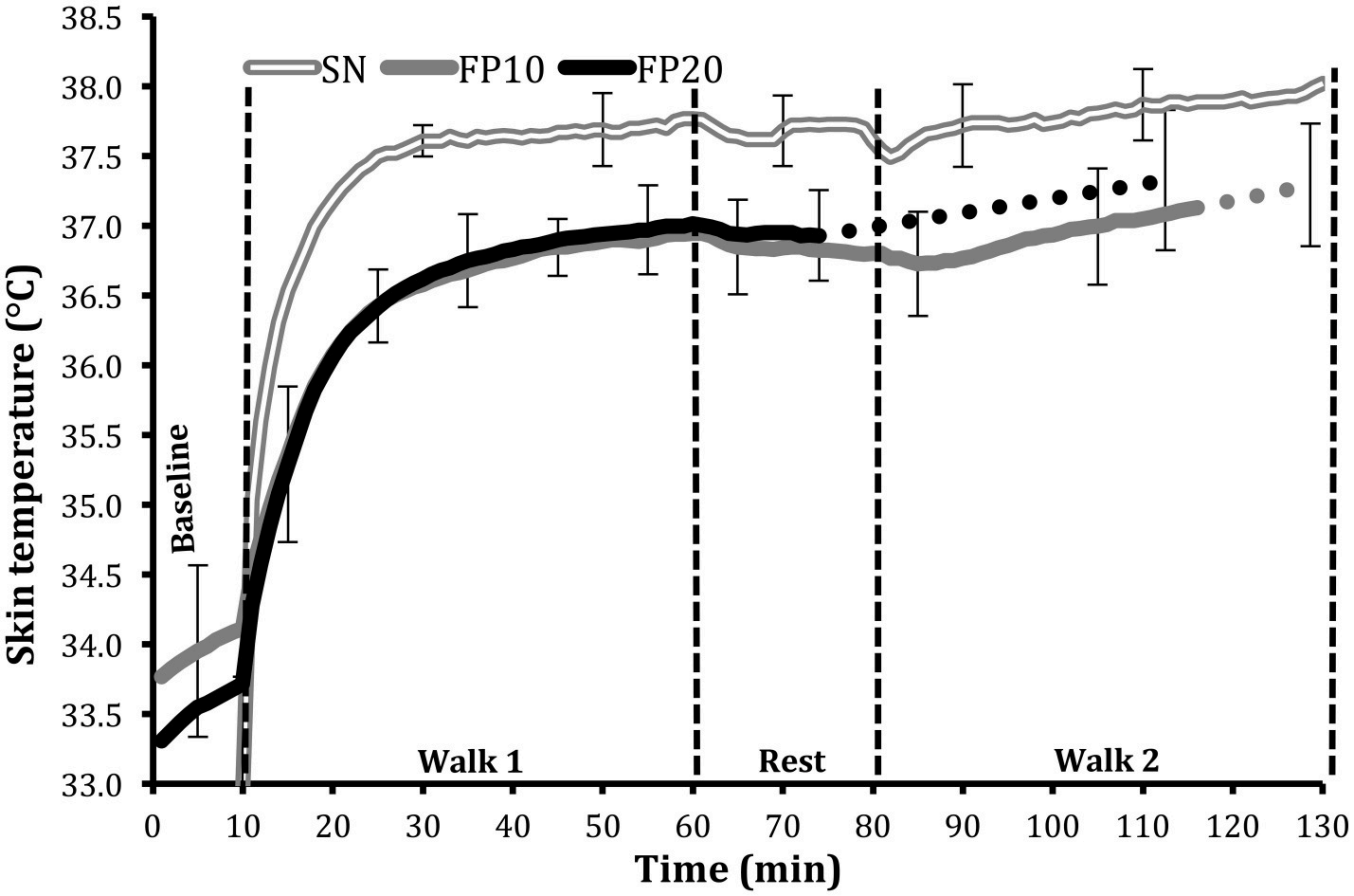
Mean skin temperature (°C) ± *SD* during the full protective equipment trials (FP), performed at 10% (FP10), and 20% (FP20) ambient relative humidity, and during semi-nude condition (SN). For the two conditions where subjects terminated the trial prematurely (FP10 and FP20), the last data point for these subjects was averaged with the final data point, obtained from the subjects completing the trial. The solid line presents all 10 subjects' data whereas the dotted lines denote interpolations of the remaining portions of the responses up to the final data point.

The first 65 min of the trial indicated higher measured Tsk in SN (37.4 ± 0.2°C), compared to FP10 (36.5 ± 0.2°C; *p* < 0.001), and FP20 (36.6 ± 0.3°C; *p* < 0.001). The same was also observed at the final time point (Figure 3) with Tsk of 37.9 ± 0.4°C in SN, compared to 37.1 ± 0.5°C in FP10 (*p* < 0.001), and 37.3 ± 0.5°C in FP20 (*p* < 0.01).

### Heat flux (Q, W.m^−2^)

During the first 65 min of the trial Q differed between all three experimental trials (FP10 vs. FP20: *p* < 0.05; FP10 vs. SN: *p* < 0.001; FP20 vs. SN: *p* < 0.001) attaining values of −11.3 ± 8.0 W.m^−2^ in FP10, −4.6 ± 6.9 W.m^−2^ in FP20, and −66.2 ± 7.3 W.m^−2^ in SN. At the final time point similar *Q*-values were measured in FP10 (11.8 ± 23.3 W.m^−2^) and FP20 (18.3 ± 16.2 W.m^−2^), but higher heat gain in SN (−59.5 ± 9.2 W.m^−2^; *p* < 0.001). The positive values of Q designate heat loss (i.e., heat flux from the skin surface to the surrounding ambient air), and the negative values designate heat gain (i.e., heat flux from the ambient air to the skin).

### Sweat secretion (Sw, g) and evaporation (E, g)

Subjects' weight loss was greater in FP20 (23 ± 8 g.min^−1^) compared to FP10 (16 ± 4 g.min^−1^; *p* = 0.02). SN condition did not differ compared to FP conditions (18 ± 2 g.min^−1^). Assuming that the sweat lost by the subjects, corrected for the amount of sweat absorbed by the clothing reflects the sweat evaporated, then the amount of sweat evaporated in FP conditions was identical, with 12 ± 1 g.min^−1^ of sweat evaporated in FP10, and 12 ± 2 g.min^−1^ evaporated in FP20 (*p* = 0.57). Significantly more sweat was evaporated in SN (17 ± 2 g.min^−1^), compared to FP conditions (*p* < 0.001).

### Oxygen uptake (VO_2_, ml/min) and minute ventilation (V_*E*_, ml/min)

When starting the first walk, VO_2_ was similar in FP10 (1,206 ± 98 ml/min) and FP20 (1,190 ± 104 ml/min; *p* = 0.58), whereas a higher VO_2_ was noted in SN (1,318 ± 123 ml/min; *p* < 0.001). VO_2_ tended to increase with time, in particular in the FP trials, and the final values indicated no difference between experimental conditions (1,375 ± 135 ml/min in FP10, 1,426 ± 131 ml/min in FP20, and 1,390 ± 143 ml/min in SN). V_E_ was identical in FP10 and FP20 (27 ± 3 L/min) when starting the first walk, but higher in SN (30 ± 4 L/min) compared to both FP conditions (*p* = 0.02). The final values indicated higher V_*E*_ in FP20 with 39 ± 8 L/min, compared to FP10 with 33 ± 5 L/min (*p* = 0.009) and SN with 34 ± 4 L/min (*p* = 0.01).

### Subjective ratings of thermal comfort (TC), wear comfort (WC), thermal sensation (TS), and perceived exertion (RPE)

Subjects rated TC, WC, TS, and RPE similarly in FP10, FP20, and SN during the first walk. During the rest period, they rated TC as better in FP10, compared to FP20 (Table 2; *p* = 0.03). WC was better in FP10 (*p* = 0.02), and SN (*p* = 0.03), when compared to FP20. The ratings of TS and RPE were lower in SN, compared to FP20 (*p* = 0.01). The final time point also indicated reduced TC and WC and increased TS and RPE in FP20, when compared to FP10 and SN (*p* < 0.05). The differences between FP10 and SN were evident only for the TS at the final time point, with subjects reporting of being “hotter” in FP10 than in SN (*p* = 0.01).

**Table 2:** Median (range) ratings of thermal comfort (TC), wear comfort (WC), thermal sensation (TS), and perceived exertion (RPE) during the first walk and rest and at the final time point in all experimental conditions.

	**First walk and rest**			**Final end point**		
	**FP10**	**FP20**	**NC**	**FP10**	**FP20**	**NC**
TC	1.1 (3.0)	1.8 (3.0)	1.3 (3.0)	1.5 (2.5)	2.3 (2.5)	1.3 (3.0)
WC	1.1 (2.5)	1.8 (3.0)	1.1 (2.8)	2.0 (2.5)	2.8 (2.5)	1.5 (2.5)
TS	1.5 (2.0)	2.0 (2.0)	1.75 (2.0)	2.0 (2.0)	3.0 (1.0)	1.5 (2.0)
RPE	12.0 (9.0)	14.0 (8.5)	11.8 (7.5)	14.0 (6.0)	16.0 (9.0)	12.5 (6.0)

## Discussion

The results from the present study indicate that a lower ambient relative humidity (i.e., 10% compared to 20% at 45°C) improves evaporative cooling, as reflected by lower core temperature, heart rate, expired volume, in the improved ratings of thermal comfort, temperature sensation, and in the reduction of the perceived exertion. Some of the physiological and subjective responses observed in the SN (semi-nude at 20% ambient RH) trial were more comparable to the responses observed in the FP10 (full protective equipment trial) trial. For example, the rectal temperature response, indicative of the heat loss/gain balance, was similar for the SN and FP10 conditions, both being significantly lower than the response observed in FP20. This implies that the removal of clothing in 20% RH improves heat loss such that heat gain is similar to that in the clothed (FP) condition at 10% RH.

Despite the significantly greater rate of sweating in FP20 compared to FP10, the evaporative heat loss was similar in the FP conditions. In the SN condition the sweat rate was similar to the FP conditions, but evaporative heat loss was substantially greater. It should be emphasized that in the SN condition, there was a minimal clothing barrier, and the sweat loss determined by the loss in subjects' mass reflected the sweat evaporated from the skin. In the FP conditions, the sweat loss corrected for the amount of sweat absorbed by the clothing does not reflect the sweat loss by evaporation from the skin, but also from the clothing. Whereas, the former impacts significantly to heat loss, the contribution of the latter may be negligible. Regardless, it is not possible from the present measurements to assign the proportion of sweat evaporated directly from the skin, and that evaporated from the clothing after it had been absorbed by the clothing. Similarly, we did not account for any sweat that may have dripped onto the floor. Although minimal, this amount contributes to the error in the estimation of evaporative heat loss in such studies.

### The influence of humidity on physiological responses to same walking speed

Several physiological responses such as rectal and skin temperature, heart rate, and pulmonary ventilation did not differ significantly between trials during the initial portions of the experiment, but exhibited substantial inter-condition differences at the trial end-point. This suggests that a 10% unit difference in ambient relative humidity will affect physiological responses during long-duration, but not necessarily short-duration, exercise in the heat. The minute effects during short-duration exposures could be a result of wearing personal protective clothing, which can protect the soldier from the heat in the early stages, especially if clothing is conditioned in cooler ambient, but reduces the evaporation in the further stages (Barwood et al., 2009). Low levels of ambient relative humidity become beneficial once a person starts producing enough sweat to allow proper heat dissipation if not prevented by protective clothing (Aoyagi et al., 1998).

As noted above, the striking difference between the two humidity conditions became apparent during, and after the 20 min rest period between the 1st and 2nd walks (Figure 2). As reflected in the heart rate responses, the FP20 condition afforded the least recovery, compared to the FP10, and SN conditions. The responses in the second walk period were dependent on subjects' status at the end of the recovery. The observed cumulative effect was least in the SN condition, and most in the FP20 condition.

### Influence of humidity on subjective ratings of temperature and work

The majority of subjects in the present study reported improved thermal comfort, sensation, and exertion at the final time point in FP10 compared to FP20, which was also reflected in some physiological responses, suggesting subjects could perceive the 10% unit difference in ambient relative humidity. In some physiological (Tre and V_*E*_ at the final time point) as well as subjective rating responses (TC, WC, and RPE at the final time point), SN and FP10 were actually comparable and significantly better than in FP20. Nine subjects completed the FP10 trial (one subject requested termination due to shoulder pain), whereas only six were able to complete the FP20 trial (four subjects terminated the trial due to heat strain), which demonstrates that the difference in the partial pressure of water vapor gradient between the skin and ambient air in the FP20 and FP10 trials significantly affected physiological and perceptual responses. Whether these improvements as a result of the 10% unit difference in ambient relative humidity would also be reflected in cognitive performance cannot be discerned from the present study. On the basis of evidence of recent studies, such an effect of cognitive performance would not be anticipated. Namely, Caldwell et al. (2012), reported that cognitive functions are not affected by a 2-h exposure to hot and dry ambient conditions while wearing biological and chemical protective clothing and performing low intensity exercise, although rectal temperature and heart rate were significantly elevated. Similarly, Schlader et al. (2016) reported that a 60-min exposure to hot and humid ambient conditions while wearing encapsulating personal protective equipment during exercise does not affect risk-taking behavior.

### Impact of clothing on performance in hot and humid environments

The impact of clothing in the three trials (SN, FP10, FP20) is clearly evident from the SN trial, where subjects' Tsk was significantly higher throughout the trial due to no barrier between the skin and the environment. As observed from the heat flux values, subjects were gaining heat from the environment, which was much hotter than the skin surface. On the other hand, with no barrier between the body and the environment, evaporation was significantly greater in the SN condition, reflected in lower Tre and HR at the final time point. During the first walk, the respiratory responses, including oxygen uptake, were greater in SN, and this is most likely due to reduced mechanical efficiency consequent to all the weight being carried in the backpack (~35 kg), rather than evenly distributed as in the FP conditions. At the final time point, by contrast, oxygen uptake were similar in all trials, which might suggest that at this point the lower energy expenditure in the FP conditions, resulting from advantageous load distribution and thus a better walking economy (Smoljanić et al., 2014), was overcome by increased energy costs resulting from hyperkinetic circulation and, in FP20, from exaggerated exercise hyperpnea.

Thus, in addition to the thermal factors that influence the rate of sweating, and thus skin wettedness, there are many non-thermal factors (Mekjavic and Eiken, 2006) that may also affect sweating, and thus evaporative heat loss. Of these some are physiological, i.e., dehydration, heat adaptation, level of training, age, etc., and others non-physiological, such as protective clothing. In conditions, where subjects wear protective clothing, it is the partial pressure of water vapor within the microclimate that dictates the rate of evaporation from the skin. Furthermore, it is the design of the clothing that determines the ventilation of the clothing microenvironment, and consequently the rate of evaporation. The critical relative humidity will therefore depend on the ambient conditions, and the conditions established within the clothing microenvironment by the protective clothing.

### Heat loss strategies

The necessity of wearing protective clothing and/or performing physical activity in hot ambient conditions raises the question of different approaches of coping with heat stress. This is becoming an increasing problem in industry during periods of heat waves, which have become more frequent, greater in magnitude, and longer in duration as a consequence of global warming. A number of studies have investigated the benefits of different cooling techniques in such conditions (Hadid et al., 2008; Barwood et al., 2009; Caldwell et al., 2012; Davey et al., 2013; Ciuha et al., 2016), as well as heat acclimatization as part of adaptation to heat (Houmard et al., 1990; Gill and Sleivert, 2001; Machado-Moreira et al., 2005), accounting for the effects of heat on physical performance (Mohr et al., 2012; Fogarty et al., 2015; Veneroso et al., 2015). However, it is not only ambient temperature that affects the performance, but also the level of ambient relative humidity, which may add an extra burden. Therefore, it is crucial to study these two ambient factors together (Alahmer et al., 2011; Maughan et al., 2012; Schlader et al., 2016). Future strategies to mitigate heat strain in workers should also explore the possibility of reducing ambient relative humidity.

In real-life scenarios, workers are exposed to a variety of ambient temperatures and relative humidities. The aim of the present study was to assess, if a reduction in ambient relative humidity at the lower end of the humidity scale significantly affects physiological responses during load carriage at an ambient temperature of 45°C. Assuming a skin temperature of 37°C and above, the difference in the partial pressure of water vapor between the skin and ambient air will decrease with increasing ambient relative humidity, and thus the drive for evaporation will also be reduced.

### Limitations and study considerations

In the present study, only 6 of the 10 subjects were able to complete the trial with higher ambient relative humidity. The analysis therefore included only 65 min of experimental trials and the final time point. Since all subjects which terminated the trial early showed signs of heat exhaustion, this nevertheless presents valuable data regarding wearing protective clothing in hot conditions.

SN was introduced in the study as a Control trial and was performed at 20% RH. As such this is a Control condition for the FP20 trial, which was performed at the same ambient RH, and not necessarily for the FP10 trial, which was conducted at 10% ambient RH. Ideally, another Control trial should have been performed at 10% ambient RH. This would have impacted on the acclimation to the conditions of the test.

For a given subject, the experimental trials were separated by a minimum of 3 days, and the order of the trials was randomized. Nevertheless, the possible effect of acclimation cannot be neglected, although any significant impact on the study outcome is unlikely.

## Ethics statement

This study was carried out in accordance with the recommendations of the Republic of Slovenia National Medical Ethics Committee with written informed consent from all subjects. All subjects gave written informed consent in accordance with the Declaration of Helsinki.

## Author contributions

IM and OE initiated the study. UC and MG conducted the experimental trials, assisted by IM and OE. All authors were involved in the analysis and interpretation of the data. IM and UC drafted the manuscript, which was revised by OE and MG.

### Conflict of interest statement

The authors declare that the research was conducted in the absence of any commercial or financial relationships that could be construed as a potential conflict of interest.
